# Repeatability of Optical Coherence Tomography Angiography in Uveitic Eyes

**DOI:** 10.1167/tvst.8.6.17

**Published:** 2019-11-15

**Authors:** Sonny Caplash, Shilpa Kodati, Shuk Kei Cheng, Marib Akanda, Susan Vitale, Ian Thompson, Sapna Gangaputra, H. Nida Sen

**Affiliations:** 1 National Eye Institute, National Institutes of Health, Bethesda, MD, USA; 2 Baylor College of Medicine, Cullen Eye Institute, Houston, TX, USA; 3 Vanderbilt Eye Institute, Nashville, TN

**Keywords:** uveitis, OCTA, repeatability

## Abstract

**Purpose:**

To investigate the intravisit repeatability of optical coherence tomography angiography (OCTA) in a cohort of uveitis patients.

**Methods:**

One hundred ten patients were imaged twice per eye, per visit, using the Zeiss Cirrus HD-OCT Model 5000 device. To calculate choriocapillaris flow void area (CC FV) 6 × 6-mm images were used, and 3 × 3-mm images were used to calculate vessel density (VD) and the foveal avascular zone area (FAZ) of the superficial capillary plexus (SCP) and deep capillary plexus (DCP). Repeatability was measured using Bland-Altman analyses and intraclass correlation coefficients (ICC) with associated coefficient of variation (CV).

**Results:**

The level of intravisit repeatability differed across indices ranging from moderate to excellent. CC FV had the highest intravisit repeatability with an ICC of 0.980 (95%CI, 0.966–0.989), a CV of 15.9% and Bland-Altman limits of agreement from −0.398 to 0.411 mm^2^. DCP FAZ had the lowest intravisit repeatability with an ICC of 0.677 (95%CI, 0.510–0.796), a CV of 17.4% and Bland-Altman limits of agreement from −0.395 to −0.355 mm^2^. Intraoperator repeatability was excellent across all indices.

**Conclusions:**

This study demonstrates that OCTA is a reliable tool to quantitatively assess specific indices of vascular structure in uveitis patients with good intravisit repeatability. However, the range of variability for each index should be taken into account when evaluating clinically meaningful changes.

**Translational Relevance:**

The repeatability of the metrics we have described has implications in supporting the development of OCTA-derived quantitative assessments of the retinal and choroidal vasculature in uveitis patients as potential imaging biomarkers.

## Introduction

Optical coherence tomography angiography (OCTA) is an emerging noninvasive technology that allows for visualization of flow in retinal and choroidal blood vessels.^1^ Fluorescein angiography and indocyanine green have been the mainstay for evaluating retinal and choroidal vasculature, respectively, in a diverse spectrum of retinal and uveitic diseases.^2–19^ While providing a relatively high-resolution picture, both imaging modalities are limited by their use of an intravenously administered dye.

OCTA is a functional extension of OCT and provides a noninvasive method to image the vasculature of eye. OCTA can provide an instant, depth-encoded visualization of the retinal vasculature and choriocapillaris. OCTA uses the same low-coherence light to identify dynamic structures of the eye, specifically the vasculature.^1^ Vasculature here is defined as a dynamic structure because as separate red blood cells move through vessels, consecutive B-scans will produce different signal patterns due to differences in red blood cell location at the time of each scan. This differential scattering is analyzed by the OCTA software to generate a map of the vasculature.^1,20^ Built-in segmentation algorithms allow the software to generate vasculature maps of various depths of the eye, thus enabling separate visualization of the superficial retinal capillary plexuses (SCP) and deep retinal capillary plexuses (DCP), as well as the choriocapillaris.^1,20,21^

OCTA has been used to qualitatively and quantitatively evaluate the retinal vasculature and choriocapillaris in both retinal and uveitic diseases.^21–25^ Quantitative measures that have been described include foveal avascular zone (FAZ) area and vessel density (VD) of both the SCP and DCP, as well as choriocapillaris total flow void area (CC FV).

As with any new technology, the repeatability of OCTA is vital to its clinical use and interpretation. Although various reports have examined the variability of OCTA quantitative measurements, the majority of reports are from healthy controls. There is a paucity of data regarding variability in uveitis patients.^26–31^ The purpose of this study was to investigate the repeatability of quantitative measurements derived from OCTA images from a cohort of uveitis patients.

## Methods

### Study Design

Patient images obtained prospectively under a clinical research protocol from January 2015 to March 2018 were analyzed. All patients were enrolled in a standardized clinical protocol, approved by the institutional review board, and consented appropriately. The study adhered to the tenets of the Declaration of Helsinki.

A total of 110 adult patients were enrolled. After excluding images with poor signal intensity or motion artifact, 85 eyes of 85 patients were included in the analyses. In bilateral cases, the right eye of each patient was included in the analyses. Images were acquired using the AngioPlex OCTA software on a Zeiss Cirrus HD-OCT (AngioPlex, CIRRUS HD-OCT model 5000; Carl Zeiss Meditec, Inc., Dublin, OH), which uses a mean value projection to produce en face images.^32,33^ Each patient was scanned twice per eye by the same technician using the same device at each visit. Patients were not repositioned at the headrest between scans and an interval of 30 seconds to 1 minute was taken between consecutive scans in a single visit. Each image was evaluated for horizontal motion artifacts and signal intensity. To objectively determine exclusion criteria, the difference between intravisit quantitative measurements were plotted against image quality indices, including average signal intensity of the two images and the level of horizontal motion artifact. Following this analysis, the image library was then refined to exclude images with a high degree of motion artifacts, floaters (evidenced by flow voids that were relatively larger and not present in consecutive scan), and low signal intensity (<7/10 signal intensity). Patients were not excluded on the basis of clinical activity. Subsequent statistical analysis was performed on these images (*N* = 85 participants, of which 7 were clinically active) to determine intravisit and intraoperator variability. Images were analyzed for SCP FAZ, SCP VD, DCP FAZ, DCP VD, and CC FV by using investigator-generated algorithms.

### Image Processing Algorithm

Each of the steps outlined below were performed using investigator-generated algorithms.

#### Choriocapillaris Flow Void Calculation

For choriocapillaris analysis, 6 × 6-mm^2^ OCTA images were acquired. Automated segmentation was used to generate images of the superficial retinal layer, avascular outer retinal layer, and choriocapillaris. These images were then exported from AngioPlex for standardization and analysis in ImageJ, an open source java-based image processing program (National Institutes of Health, Bethesda, MD).

Images were first standardized to have a normal distribution of pixel intensity, in order to make all images have a comparable pixel-intensity distribution. Standardization of images was performed by brightness histogram contrast stretching; a central square at 50% of the image size was used as reference to normalize the intensity histogram. The choriocapillaris slab is susceptible to projection artifacts from the superficial retinal plexus, which can be falsely interpreted as flow deficits due to their inherent lower signal intensity.^34^ Therefore, following standardization, projection artifacts from the superficial retinal plexus were minimized by first binarizing the corresponding superficial retinal plexus image and overlaying it upon the choriocapillaris image to align projection artifacts in the choriocapillaris with specific vessels from the superficial retinal plexus. Following alignment of these two images, the algorithm increased intensity values in regions identified as artifact through overlay, thereby reducing the quantitative impact of the artifacts in further analysis.

Following standardization and removal of projection artifacts, the two consecutive scans from the same visit were aligned against each other using the TrakEM tool on ImageJ. Images were then manually cropped to include only the overlapping area between the two intravisit images and were subsequently exported for threshold analysis.

**Figure 1:**
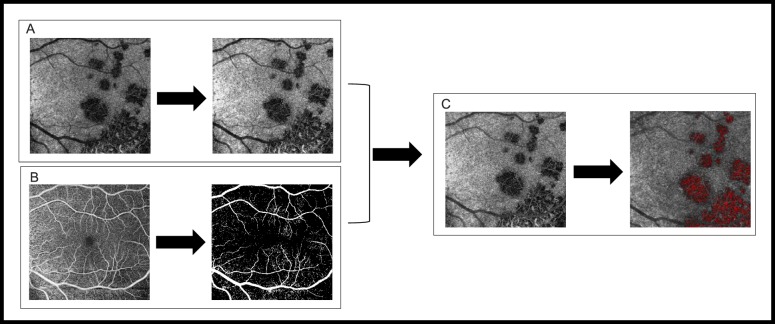
Shows the steps by which an investigator-generated algorithm calculates CC FV. All image slabs were generated by automated segmentation from Angioplex OCTA software on a Zeiss Cirrus HD-OCT Model 5000 device. (A) This panel shows the choriocapillaris slab before and after image standardization via brightness histogram contrast stretching. (B) This panel shows the SCP slab before and after binarization. (C) The final images in panels A and B are overlaid to remove projection artifacts and generate the first image in panel C. The choriocapillaris image then undergoes alignment and cropping, followed by FV quantification using the pixel intensity of the avascular slab as a threshold for measurement. All pixels with intensities at or below the defined threshold are highlighted in red and quantified as shown in panel C. It is important to note that binarization and projection artifact removal do not visually remove shadows generated on the choriocapillaris by the overlying SCP.

To quantify total flow void area, a threshold for the pixel intensity of flow deficit was defined using the corresponding avascular image slab. The threshold was determined using the average pixel intensity and its standard deviation in the following equation: $\newcommand{\bialpha}{\boldsymbol{\alpha}}\newcommand{\bibeta}{\boldsymbol{\beta}}\newcommand{\bigamma}{\boldsymbol{\gamma}}\newcommand{\bidelta}{\boldsymbol{\delta}}\newcommand{\bivarepsilon}{\boldsymbol{\varepsilon}}\newcommand{\bizeta}{\boldsymbol{\zeta}}\newcommand{\bieta}{\boldsymbol{\eta}}\newcommand{\bitheta}{\boldsymbol{\theta}}\newcommand{\biiota}{\boldsymbol{\iota}}\newcommand{\bikappa}{\boldsymbol{\kappa}}\newcommand{\bilambda}{\boldsymbol{\lambda}}\newcommand{\bimu}{\boldsymbol{\mu}}\newcommand{\binu}{\boldsymbol{\nu}}\newcommand{\bixi}{\boldsymbol{\xi}}\newcommand{\biomicron}{\boldsymbol{\micron}}\newcommand{\bipi}{\boldsymbol{\pi}}\newcommand{\birho}{\boldsymbol{\rho}}\newcommand{\bisigma}{\boldsymbol{\sigma}}\newcommand{\bitau}{\boldsymbol{\tau}}\newcommand{\biupsilon}{\boldsymbol{\upsilon}}\newcommand{\biphi}{\boldsymbol{\phi}}\newcommand{\bichi}{\boldsymbol{\chi}}\newcommand{\bipsi}{\boldsymbol{\psi}}\newcommand{\biomega}{\boldsymbol{\omega}}T = {A_p} + 1.96\left( {{S_d}} \right)$, where *T* is the flow deficit pixel intensity threshold, *A_p_* is the average pixel intensity of the image and *S_d_* is the standard deviation among individual pixel intensities in the image. Subsequently, regions with pixel intensities at or below the defined threshold were highlighted and measured to determine the total FV in the choriocapillaris images (Fig. 1).

#### Vessel Density Analysis

For analysis, 3 × 3-mm^2^ OCTA images were acquired. Automated segmentation was used to generate images of the SCP and DCP. These images were then exported from AngioPlex for standardization in ImageJ.

Images were standardized in the same process detailed above. Of note, in DCP images, the projection artifacts from the overlying SCP were not removed. Following standardization, images were binarized using Otsu global threshold algorithm to create an image with the vessels in black and nonvessel areas of the OCTA as a white background. Binarization allows for quantification of total vessel area (TVA) through summation of all black pixels. The VD was derived by division of the TVA by the total image area as follows: VD = (TVA)/(total image area). Following binarization and summation of black pixels, the FAZ was manually delineated by the user.

### Statistical Analysis

Intravisit and intraoperator repeatability were calculated for all quantitative measurements by using Bland-Altman modeling.^35^ For each pair of values, the averages of the two measurements were plotted against their differences and the coefficient of repeatability (CR) was calculated. The CR was defined as 1.96 multiplied by the standard deviation (SD) of the differences.

Intraclass correlation coefficients (ICC) were also calculated for each set of variabilities. A two-way, mixed-effect, consistency model (ICC 3, 1) was used for ICC calculations.^36^ Pearson's correlation was used to assess the correlation between intravisit repeatability and clinical activity. IBM SPSS Statistics 25 software (IBM Corp, Armonk, NY) was used for statistical analysis. ICC is computed as ST^2^/(ST^2^ + SE^2^), where ST^2^ is variability among subjects and SE^2^ is measurement variance. Therefore, if ST^2^ is large (i.e., if there is a lot of heterogeneity among participants) ICC will be close to 1 regardless of the value of SE^2^. Therefore, the ICC may not the best method for assessing repeatability in many study designs^37^ and may inflate the repeatability assessment in heterogeneous data. The coefficient of variation (CV) is defined as the ratio of the within-person SD to the mean. It can be used to further contextualize the ICC by providing an estimate of within-subject variance.

An operator in the context of this study was defined as the person processing the images. Intravisit variability was defined as the variability in quantitative measurements between two images taken at the same visit of the same eye by the same technician using the same device and processed by one operator. Intraoperator variability was defined as the variability in quantitative measurements when one operator processed the same image on two separate occasions while being masked to the identity of each image. The primary focus of this study is to assess the intravisit repeatability; intraoperator variability was calculated to assure it did not contribute significantly to intravisit variation.

## Results

Patients analyzed in this study were categorized into two groups based on the disease process and analysis performed. The first group involved patients with diseases known or suspected to affect the choroid, referred to as the “choriocapillaris involving group” (CC), and were analyzed by quantifying total CC FV. This group included 62 patients in total and had pathologies ranging from birdshot chorioretinopathy (BCR) to Vogt-Koyanagi-Harada (VKH) syndrome. The etiologies and demographics of this group are summarized in Tables 1 and 2, respectively. After exclusion of patients with significant motion artifact or signal intensity less than seven, there were 50 total patients eligible for analysis.

**Table 1:** Summary of Uveitis Etiology in Choriocapillaris Patient Group

Pathologies in CC Patient Group	*n* (%)
BCR	21 (34)
VKH syndrome	12 (19)
White dot syndromes	24 (39)
Punctate inner choroidopathy	9 (15)
Serpiginous/ampiginous choroiditis	14 (22)
Multiple evanescent white dot syndrome	1 (2)
Idiopathic	4 (6)
Sarcoidosis	1 (2)
Total	62

**Table 2:** Demographic Data of All Tested Patients

	CC Patients	All VA Patients	Panuveitis	Intermediate Uveitis	BCR
*N*	62	69	22	26	21
Mean age (range)	48.61 (19–82)	50.41 (18–88)	47.45 (18–88)	34.57 (19–73)	60.71 (32–82)
Female, %	62.9	63.7	50.0	80.7	57.1

The second group was termed the vessel analysis (VA) group and describes a cohort of patients in which OCTA images were analyzed to determine FAZ area and VD. This cohort consisted of 69 total patients; specifically, 22 panuveitis patients, 26 intermediate uveitis patients, and 21 BCR patients. The demographics of this group are included in Table 2. After exclusion of patients with significant motion artifact or signal intensity less than seven, there were 56 total patients eligible for analysis, including 19 panuveitis patients, 20 intermediate uveitis patients, and 17 BCR patients.

Prior to the repeatability analyses, an initial analysis was conducted in order to investigate the effects of intrinsic qualities of both scan acquisition and algorithm analysis on repeatability. Signal strength was not found to display a strong direct correlation with differences between repeated measurements as evidenced by a correlation coefficient (*r*^2^) of 0.250. Neither the level of avascular threshold for flow voids used for the CC FV algorithm nor the amount of cropping performed during the alignment stage of that algorithm were directly correlated with differences between repeated measures (*r*^2^ values < 0.1).

**Figure 2:**
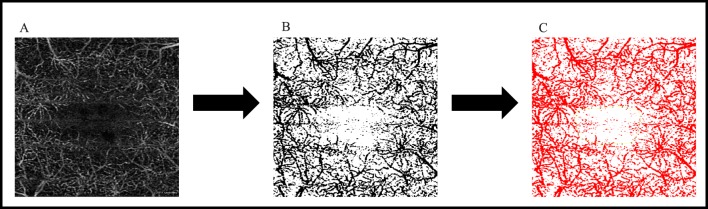
Illustrates the steps by which the algorithm calculates TVA and FAZ area in the SCP and DCP. (A) Shows the DCP of a patient, which is then binarized using Otsu global thresholding to generate (B). All black pixels are quantified and marked in red to calculate the TVA, as shown in (C). The FAZ is manually delineated on (C) to generate the FAZ area.

**Figure 3:**
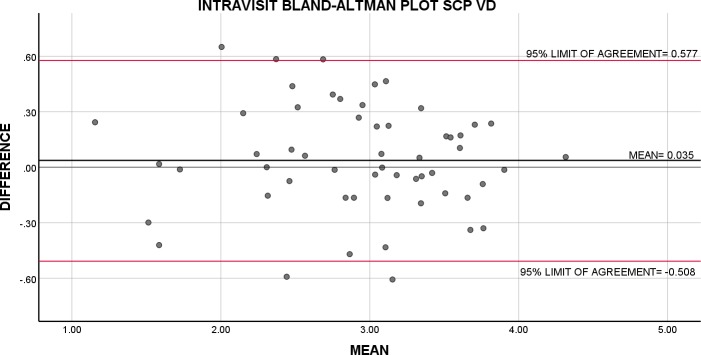
Bland-Altman plot of intravisit repeatability of VD in the SCP. The average of repeated intravisit measurements is plotted against their difference.

**Figure 4:**
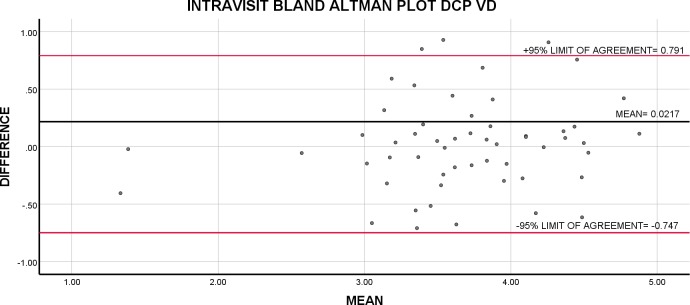
Bland-Altman plot of intravisit repeatability of VD in the DCP. The average of repeated intravisit measurements is plotted against their difference.

Intravisit Bland-Altman analysis showed the highest repeatability for SCP VD and DCP VD followed by CC FV with 95% limits of agreement ranging from −0.508 to 0.577 for SCP VD, −0.748 to 0.791 for DCP VD, and −0.398 to 0.411 for CC FV. Repeatability for both SCP and DCP FAZ were poor (Table 3). Because Bland-Altman analysis does not generate a CV, we used CR as a percentage of average value for all tested subjects across all indices as a measure of variability (Table 3). The variability for all indices ranged from 19.1% to 64.1%. The intravisit CR as a percentage of average value for all tested subjects was lowest for SCP VD and DCP VD at 19.1% and 21.0%, respectively; demonstrating 95% of all repeated measurements fall within a margin that is approximately 20% of the average measurement for SCP VD and DCP VD (Figs. 1–4).

**Table 3:** Bland Altman Analyses of Intravisit Repeatability

Quantitative Variable	*n*	Bias	95% Limits of Agreement	*P* Value	Intravisit CR, mm^2^	Average Value, mm^2^ [SD]	CR/ Average Value
CC Flow Void Area	50	−0.003	(−0.398, 0.411)	0.905	0.401	1.16 [1.88]	0.346
SCP FAZ	56	0.039	(−0.234, 0.294)	0.099	0.264	0.412 [0.252]	0.641
DCP FAZ	56	−0.019	(−0.395, 0.355)	0.605	0.374	0.741 [0.214]	0.505
SCP VD	56	0.035	(−0.508, 0.577)	0.365	0.561	2.92 [0.663]	0.191
DCP VD	56	0.0216	(−0.748, 0.791)	0.682	0.769	3.66 [0.670]	0.210

ICC for intravisit measurements were very good across all variables (ICC > 0.84) except DCP FAZ (ICC = 0.68). CC FV, SCP, and DCP VD displayed the highest intravisit ICC across all analyzed metrics. ICC for CC FV intravisit measurements ranged from 0.966 to 0.989 across all tested subjects and SCP VD intravisit measurements similarly ranged from 0.853 to 0.947. The lowest intravisit ICC was seen with DCP FAZ, which ranged from 0.510 to 0.796 (Table 4).

**Table 4:** ICC and CV for Intravisit Repeatability

Quantitative Variable	*n*	ICC (95%CI)	CV
Flow Void Area	53	0.980 (0.966, 0.989)	15.9%
SCP FAZ	64	0.867 (0.785, 0.918)	21.6%
DCP FAZ	64	0.677 (0.510, 0.796)	17.4%
SCP VD	64	0.911 (0.853, 0.947)	7.6%
DCP VD	64	0.842 (0.743, 0.905)	8.1%

The ICCs of FAZ and VD values were compared among the subgroups as follows: BCR, panuveitis, and intermediate uveitis and showed no significant differences in ICC values across all indices for different types of uveitides (Supplementary Table S1). In order to elucidate confounders in observed levels of intravisit variability described above, intraoperator repeatability was measured for all indices and showed low CV and high ICCs for all indices with lowest repeatability being associated with FAZ indices. (Supplementary Tables S2 and S3). Of 85 patients (85 eyes) included in this study, seven (8%) were active at the time of image acquisition. There was no significant correlation between the repeatability measures and the activity of uveitis at the time of measurement (Supplementary Table S4).

## Discussion

This study investigated the intravisit and intraoperator repeatability of OCTA, as well as possible indices of image quality that may impact repeatability in a cohort of uveitis patients. Given the increasing use of quantitative metrics in OCTA, there is an emerging need for data on the repeatability of these metrics.

Our results show excellent intravisit repeatability for total CC FV, TVA of the superficial retinal plexus, and the FAZ area of the superficial retinal plexus. VD measurements of the DCP showed good repeatability and FAZ measurements of the DCP showed moderate intravisit repeatability. Intraoperator repeatability was excellent across all variables and is unlikely to significantly contribute to variability observed.

Comparing our data with the existing literature examining repeatability and reliability of OCTA in healthy subjects yielded mixed results. Carpineto et al.^26^ examined a group of 60 healthy volunteers with a high-resolution spectral-domain OCT (SD-OCT) XR Avanti using a split-spectrum amplitude decorrelation angiography algorithm. ICC values ranged from 0.995 to 0.999, which were comparatively higher than in our cohort of uveitis patients with SCP FAZ ICC ranging from 0.785 to 0.918. Chen et al.^27^ used the RTVue XR Avanti system to image 50 healthy individuals twice in the right eye in the same visit. FAZ and VD measurements of the SCP were machine-generated and subsequent statistical analysis yielded CRs of 0.052 and 0.099, respectively. In comparison to CRs yielded by our study for SCP FAZ and VD, 0.264 and 0.561 respectively, the analysis of healthy volunteers by Chen et al.^27^ demonstrated higher repeatability. Fang et al.^38^ used a DRI OCT Triton to image 33 subjects aged 18 to 40 and reported intrasession ICCs of 0.996 and 0.853 for SCP FAZ and VD, respectively. This is comparatively higher as compared with SCP FAZ and relatively equivocal to SCP VD resulted from our investigation. Zhang et al.^39^ recently published data regarding intravisit repeatability of choriocapillaris flow voids using the PLEX Elite 9000 SS-OCTA system. An analysis of 20 healthy subjects yielded a CV 5.38%, approximately one-third of the calculated CV generated by this study.^39^ Comparison with similar studies reveals over lower repeatability of CC FV and SCP FAZ in our cohort of uveitis patients as compared with healthy control. Comparison of SCP VD in our cohort of uveitis patients with healthy subjects revealed examples of both comparable repeatability and decreased repeatability.

Closer analysis of ICC data reveals several trends. First, measurements of the DCP have lower intravisit ICC values than measurements of the SCP. This may be due to the intrinsic scan acquisition of the DCP; vessels are observed to be less defined, otherwise read as having lower contrast. A similar finding was observed by Fenner et al.^40^ in a cohort of healthy subjects. Because our algorithm relies on binarization of the image using specific pixel intensity threshold values, decreased contrast in overall scan acquisition makes artifacts less distinguishable, and therefore more likely to be included after binarization. This inclusion may allow artifacts to have a larger impact on the DCP image, and therefore VD and FAZ measurements. Second, FAZ measurements had overall increased variability, which is expected due to manual delineation involved. However, intraoperator repeatability measures of FAZ measurements did not entirely explain intravisit variability, which was relatively higher compared with other indices. Nevertheless, ICC values were still in a range deemed statistically reliable. Examination of studies looking at healthy controls did not show a similar pattern, though most of those investigations used machine-generated calculations of FAZ area. Third, and last, there was no statistically significant correlation between activity of disease or type of uveitis with repeatability indices. Similarly, a previous study by Kim et al.^41^ investigated skeleton density, VD, fractal dimension, and vessel diameter index in 94 healthy eyes and 81 uveitic eyes using a prototype Cirrus SD-OCTA and indicated no relationship between type of uveitis and repeatability and implied no difference in repeatability between healthy and uveitic eyes.

Our study looked at two statistical models, the ICC and the Bland-Altman model, both of which look at repeatability. While the ICC data shows high levels of repeatability, the Bland-Altman model does not show equivocally strong data. Intravisit FV, for example, had an ICC of 0.980, whereas it had a coefficient repeatability of 0.401 mm^2^, a value that delineates the margin by which 95% of repeat measurements “agree.” This CR, in context, is roughly 35% of average FV, implying mild repeatability. The trend holds true across all variables with coefficients of repeatability that were as high as 64.1% (SCP FAZ) and 38.8% (DCP FAZ) of their average measurements with corresponding ICCs of 0.868 and 0.751. The dichotomy reveals a possible bias that may be introduced with ICC, which can be roughly translated as the variability between two repeat measurements within a patient divided by the variability among all measurements within a patient cohort. Relatively low variability within the patient cohort can therefore artificially deflate the ICC. The Bland-Altman model instead is relatively less biased as it is a simple plot of the difference of two repeat measurements against their average.

This study also examined the confounding (or effect modification) of horizontal motion artifacts and signal strength on the repeatability of intravisit OCTA scans. Subanalysis of our data implies that horizontal motion artifacts and signal strength likely do not significantly influence either the intravisit and intraoperator variability. This may be due to the fact that horizontal motion artifacts, with respects to area in pixels, may not represent a large percentage of the overall analyzed image. Signal strength greater than seven does not appear to have a measurable impact on the intravisit repeatability.^29^ Regarding the specific algorithms used in this study, signal strength greater than seven, excluding extremely poor scans (with no discernible anatomy), does not pose a problem because both algorithms use thresholds for calculation. The CC algorithm uses a threshold generated by the avascular zone and the FAZ/VD uses Otsu global thresholding algorithm during binarization. The threshold likely counteracts any diminished contrast generated from poor signal strength, because despite low signal, so long as relative contrasts are preserved after thresholding, consistent data can be generated. The lack of strong correlation of variability with signal strength or horizontal motion artifact coupled with low intraoperator variability, implies that any remaining variability seen in the data may in fact be variability from fixation during scan acquisition, possible physiologic variability, or the technology itself.

Our study has its limitations. We only included adult patients; therefore, our results may not be applicable to children with uveitis. The proportion of patients with active uveitis was relatively low in this cohort and our finding of lack of significant correlation between disease activity and variability needs further confirmation. This study also made use of Otsu global threshold algorithm for generation of binarized SCP and DCP images. This algorithm, in comparison to Otsu local thresholding, fails to account for nonuniform lighting of retina by the device and results in a possible reduction in visualized vessels after binarization. The strength of our study is that OCTA data were prospectively acquired in a relatively large cohort of uveitis patients in the same session, by the same technician using the same device. We used two investigator-generated algorithms. One optimizes projection artifact removal, crops and aligns images to standardized landmarks and measures flow voids in the choriocapillaris. The other binarizes images, and computes both TVA and VD in an automated fashion and FAZ in the SCP and DCP in a semiautomated fashion. Additionally, we evaluated repeatability of all metrics using two different analytic approaches knowing that ICC may artificially inflate results in homogenous cohorts. As suspected, ICC yielded better repeatability parameters than Bland-Altman. Nevertheless, for most indices we found good to excellent repeatability.

In summary, our study of a large cohort of uveitis patients indicated strong intravisit and intraoperator repeatability for VD and choroidal flow voids. We also found that signal strength and mild horizontal motion artifact may not play a strong role in the generation of quantitative output, as well as disease activity and type of uveitis. These findings can be useful in assessing the significance of changes over time; however, the range of variability for each index should be taken into account when evaluating clinically meaningful changes.

## Supplementary Material

Supplement 1

## References

[1] Coscas G, Lupidi M, Coscas F (2016). Heidelberg Spectralis optical coherence tomography angiography: technical aspects. *Dev Ophthalmol*.

[2] Sickenberg M (2001). Early detection, diagnosis and management of choroidal neovascularization in age-related macular degeneration: the role of ophthalmologists. *Ophthalmologica*.

[3] Desmettre T, Devoisselle JM, Mordon S (2000). Fluorescence properties and metabolic features of indocyanine green (ICG) as related to angiography. *Surv Ophthalmol*.

[4] Herbort CP, Cimino L, Abu El, Asrar AM (2005). Ocular vasculitis: a multidisciplinary approach. *Curr Opin Rheumatol*.

[5] Mehdipoor G, Davatchi F, Ghoreishian H, Arjmand Shabestari A (2018). Imaging manifestations of Behcet's disease: key considerations and major features. *Eur J Radiol*.

[6] Agarwal A, Mahajan S, Khairallah M, Mahendradas P, Gupta A, Gupta V (2017). Multimodal imaging in ocular tuberculosis. *Ocul Immunol Inflamm*.

[7] Mahajan S, Invernizzi A, Agrawal R, Biswas J, Rao NA, Gupta V (2017). multimodal imaging in sympathetic ophthalmia. *Ocul Immunol Inflamm*.

[8] Wolfensberger TJ, Herbort CP (1999). Indocyanine green angiographic features in ocular sarcoidosis. *Ophthalmology*.

[9] Silpa-Archa S, Silpa-Archa N, Preble JM, Foster CS (2016). Vogt-Koyanagi-Harada syndrome: perspectives for immunogenetics, multimodal imaging, and therapeutic options. *Autoimmun Rev.* Aug.

[10] Raven ML, Ringeisen AL, Yonekawa Y, Stem MS, Faia LJ, Gottlieb JL (2017). Multi-modal imaging and anatomic classification of the white dot syndromes. *Int J Retina Vitreous*.

[11] Yang SJ, Salek S, Rosenbaum JT (2017). Ocular sarcoidosis: new diagnostic modalities and treatment. *Curr Opin Pulm Med*.

[12] Nazari Khanamiri H, Rao NA (2013). Serpiginous choroiditis and infectious multifocal serpiginoid choroiditis. *Surv Ophthalmol*.

[13] Bansal R, Gupta A, Gupta V (2012). Imaging in the diagnosis and management of serpiginous choroiditis. *Int Ophthalmol Clin.* Fall.

[14] Herbort CP (2009). Fluorescein and indocyanine green angiography for uveitis. *Middle East Afr J Ophthalmol.* Oct.

[15] Marmor MF, Ravin JG (2011). Fluorescein angiography: insight and serendipity a half century ago. *Arch Ophthalmol.* Jul.

[16] Delori FC, Dorey CK, Staurenghi G, Arend O, Goger DG, Weiter JJ (1995). In vivo fluorescence of the ocular fundus exhibits retinal pigment epithelium lipofuscin characteristics. *Invest Ophthalmol Vis Sci*.

[17] Blacharski PA (1985). Twenty-five years of fluorescein angiography. *Arch Ophthalmol*.

[18] Norton EW, Gass JD, Smith JL, Curtin VT, David NJ, Justice J (1965). Macular diseases: diagnosis. Fluorescein in the study of macular disease. *Trans Am Acad Ophthalmol Otolaryngol*.

[19] Marmor MF, Ravin JG (2011). Fluorescein angiography: Insight and serendipity a half century ago. *Arch Ophthalmol*.

[20] Kashani AH, Chen CL, Gahm JK (2017). Optical coherence tomography angiography: a comprehensive review of current methods and clinical applications. *Prog Retin Eye Res*.

[21] Pichi F, Sarraf D, Arepalli S (2017). The application of optical coherence tomography angiography in uveitis and inflammatory eye diseases. *Prog Retin Eye Res.* Jul.

[22] Hua R, Wang H (2017). Dark signals in the choroidal vasculature on optical coherence tomography angiography: an artefact or not?. *J Ophthalmol*.

[23] Wang JC, Lains I, Sobrin L, Miller JB (2017). Distinguishing white dot syndromes with patterns of choroidal hypoperfusion on optical coherence tomography angiography. *Ophthalmic Surg Lasers Imaging Retina*.

[24] Pellegrini M, Acquistapace A, Oldani M (2016). Dark atrophy: an optical coherence tomography angiography study. *Ophthalmology*.

[25] Roberts PK, Nesper PL, Goldstein DA, Fawzi AA (2018). Retinal capillary density in patients with birdshot chorioretinopathy. *Retina*.

[26] Carpineto P, Mastropasqua R, Marchini G, Toto L, Di Nicola M, Di Antonio L (2016). Reproducibility and repeatability of foveal avascular zone measurements in healthy subjects by optical coherence tomography angiography. *Br J Ophthalmol*.

[27] Chen FK, Menghini M, Hansen A, Mackey DA, Constable IJ, Sampson DM (2018). Intrasession repeatability and interocular symmetry of foveal avascular zone and retinal vessel density in OCT angiography. *Trans Vis Sci Technol*.

[28] Guo J, She X, Liu X, Sun X (2017). Repeatability and reproducibility of foveal avascular zone area measurements using AngioPlex spectral domain optical coherence tomography angiography in healthy subjects. *Ophthalmologica*.

[29] Lei J, Durbin MK, Shi Y (2017). Repeatability and reproducibility of superficial macular retinal vessel density measurements using optical coherence tomography angiography en face images. *JAMA Ophthalmol*.

[30] Mastropasqua R, Toto L, Mattei PA (2017). Reproducibility and repeatability of foveal avascular zone area measurements using swept-source optical coherence tomography angiography in healthy subjects. *Eur J Ophthalmol*.

[31] Zhang Z, Huang X, Meng X (2017). In vivo assessment of macula in eyes of healthy children 8 to 16 years old using optical coherence tomography angiography. *Sci Rep*.

[32] Rosenfeld PJ, Durbin MK, Roisman L (2016). ZEISS Angioplex spectral domain optical coherence tomography angiography: technical aspects. *Dev Ophthalmol*.

[33] Huang Y, Zhang Q, Thorell MR (2014). Swept-source OCT angiography of the retinal vasculature using intensity differentiation-based optical microangiography algorithms. *Ophthalmic Surg Lasers Imaging Retina*.

[34] Spaide RF, Fujimoto JG, Waheed NK (2015). Image artifacts in optical coherence tomography angiography. *Retina*.

[35] Conant JL, Powers J, Sharp G, Mead PS, Nelson CA (2018). Lyme disease testing in a high-incidence state: clinician knowledge and patterns. *Am J Clin Pathol*.

[36] Koo TK, Li MY (2016). A guideline of selecting and reporting intraclass correlation coefficients for reliability research. *J Chiropr Med*.

[37] Bland JM, Altman DG (1990). A note on the use of the intraclass correlation coefficient in the evaluation of agreement between two methods of measurement. *Comput Biol Med*.

[38] Fang D, Tang FY, Huang H, Cheung CY, Chen H (2019). Repeatability, interocular correlation and agreement of quantitative swept-source optical coherence tomography angiography macular metrics in healthy subjects. *Br J Ophthalmol*.

[39] Zhang Q, Zheng F, Motulsky EH (2018). A novel strategy for quantifying choriocapillaris flow voids using swept-source OCT angiography. *Invest Ophthalmol Vis Sci*.

[40] Fenner BJ, Tan GSW, Tan ACS, Yeo IYS, Wong TY, Cheung GCM (2018). Identification of imaging features that determine quality and repeatability of retinal capillary plexus density measurements in OCT angiography. *Br J Ophthalmol*.

[41] Kim AY, Rodger DC, Shahidzadeh A (2016). Quantifying retinal microvascular changes in uveitis using spectral-domain optical coherence tomography angiography. *Am J Ophthalmol*.

