# Supplementary material for: Repeatability of Optical Coherence Tomography Angiography in Uveitic Eyes
Source: Transl Vis Sci Technol. 2019 Nov 15;8(6):17. doi: 10.1167/tvst.8.6.17 (PMC6859831; doi:10.1167/tvst.8.6.17)
Supplement: Supplement 1 [file tvst-08-06-02_s01.pdf]

Supplementary Data

Supplemental Table 1: Intraclass correlation coefficient (ICC) for intravisit repeatability by uveitis subtype.

	ICC	95% CI	
**SCP VD**	**0.911**	**0.853**	**0.947**
PanU	0.874	0.704	0.950
IU	0.931	0.835	0.972
BCR	0.918	0.782	0.971
**DCP VD**	**0.842**	**0.743**	**0.905**
PanU	0.869	0.693	0.948
IU	0.787	0.528	0.912
BCR	0.814	0.559	0.928
**SCP FAZ**	**0.867**	**0.785**	**0.918**
PanU	0.825	0.625	0.923
IU	0.974	0.931	0.991
BCR	0.534	0.087	0.802
**DCP FAZ**	**0.677**	**0.510**	**0.796**
PanU	0.795	0.560	0.911
IU	0.831	0.603	0.933
BCR	0.770	0.457	0.913

Pan U: panuveitis, IU: intermediate uveitis, BCR: birdshot chorioretinopathy

Supplemental Table 2: Intraoperator Repeatability (Bland-Altman Analysis)

Quantitative Variable	(n)	Bias	95% Limits of Agreement	Intraoperator CR	Average Value (mm^2) [SD]	CR / Average Value
Flow Void Area (mm^2)	35	-0.026	(-0.146, 0.0923)	.119	1.399 [2.21]	.085
SCP FAZ(mm^2)	46	-0.011	(-0.073, 0.051)	.061	.407 [0.198]	.149
DCP FAZ (mm^2)	46	-0.001	(-0.232, 0.229)	.231	.728 [0.234]	.288
SCP VD (mm^2)	46	0	(0,0)	0	2.94 [0.742]	0
DCP VD (mm^2)	46	0	(0,0)	0	3.4 [0.932]	0

Supplemental Table 3: Intraoperator Repeatability (ICC Analysis)

Quantitative Variable	(n)	ICC (95% CI)
Flow Void Area (mm^2)	35	1.00 (0.999,1.00)
SCP FAZ (mm^2)	46	0.988 (0.977, 0.993)
DCP FAZ (mm^2)	46	0.882 (0.795, 0.933)
SCP VD (mm^2)	46	1.000 (1.00,1.00)
DCP VD (mm^2)	46	1.000 (1.00,1.00)

Supplemental Table 4: Pearson Correlation Coefficient of Intravisit Percent Difference and Clinical Activity

	CC FV	DCP TVA	SCP TVA	DCP FAZ	SCP FAZ
Pearson Correlation Coefficient for Activity	0.363	-0.059	-0.072	-0.032	0.052
Sig (2-tailed)	0.203	0.688	0.621	0.830	0.725
